# Listeriolysin O Is Necessary and Sufficient to Induce Autophagy during *Listeria monocytogenes* Infection

**DOI:** 10.1371/journal.pone.0008610

**Published:** 2010-01-06

**Authors:** Nicole Meyer-Morse, Jennifer R. Robbins, Chris S. Rae, Sofia N. Mochegova, Michele S. Swanson, Zijiang Zhao, Herbert W. Virgin, Daniel Portnoy

**Affiliations:** 1 Department of Molecular and Cellular Biology, University of California, Berkeley, California, United States of America; 2 Department of Biology, Xavier University, Cincinnati, Ohio, United States of America; 3 School of Public Health, University of California, Berkeley, California, United States of America; 4 Department of Microbiology and Immunology, University of Michigan Medical School, Ann Arbor, Michigan, United States of America; 5 Department of Pathology and Immunology, Washington University School of Medicine, St. Louis, Missouri, United States of America; Columbia University, United States of America

## Abstract

**Background:**

Recent studies have suggested that autophagy is utilized by cells as a protective mechanism against *Listeria monocytogenes* infection.

**Methodology/Principal Findings:**

However we find autophagy has no measurable role in vacuolar escape and intracellular growth in primary cultured bone marrow derived macrophages (BMDMs) deficient for autophagy (*atg5^−/−^*). Nevertheless, we provide evidence that the pore forming activity of the cholesterol-dependent cytolysin listeriolysin O (LLO) can induce autophagy subsequent to infection by *L. monocytogenes*. Infection of BMDMs with *L. monocytogenes* induced microtubule-associated protein light chain 3 (LC3) lipidation, consistent with autophagy activation, whereas a mutant lacking LLO did not. Infection of BMDMs that express LC3-GFP demonstrated that wild-type *L. monocytogenes* was encapsulated by LC3-GFP, consistent with autophagy activation, whereas a mutant lacking LLO was not. *Bacillus subtilis* expressing either LLO or a related cytolysin, perfringolysin O (PFO), induced LC3 colocalization and LC3 lipidation. Further, LLO-containing liposomes also recruited LC3-GFP, indicating that LLO was sufficient to induce targeted autophagy in the absence of infection. The role of autophagy had variable effects depending on the cell type assayed. In *atg5^−/−^* mouse embryonic fibroblasts, *L. monocytogenes* had a primary vacuole escape defect. However, the bacteria escaped and grew normally in *atg5^−/−^* BMDMs.

**Conclusions/Significance:**

We propose that membrane damage, such as that caused by LLO, triggers bacterial-targeted autophagy, although autophagy does not affect the fate of wild-type intracellular *L. monocytogenes* in primary BMDMs.

## Introduction

*Listeria monocytogenes* is a Gram-positive, facultative intracellular, food-borne pathogen that causes severe disease in pregnant and immunocompromised hosts [1]. *L. monocytogenes* is also an important model organism that has been used for decades to study bacterial pathogenesis, immunology and cell biology [2]–[5].

The intracellular life cycle of *L. monocytogenes* has been described in detail [1]. *L. monocytogenes* can enter either phagocytic or non-phagocytic cells, where it is initially contained in a membrane-bound vacuole that matures through the endocytic pathway. Following acidification, the bacterium escapes from a vacuole into the host cell cytosol by secreting a cholesterol-dependent pore-forming cytolysin, listeriolysin O (LLO) [6]–[8]. The precise mechanism by which LLO induces vacuolar destruction and bacterial escape into the cytosol is not completely understood.

Subsequent to infection, approximately 10% of internalized bacteria are in the cytosol as early as 10 minutes post infection while approximately 80% of the bacteria are in the cytosol after 90 minutes [9]. Once in the cytosol, *L. monocytogenes* express the bacterial protein ActA to facilitate bacterial motility and ultimately cell-to-cell spread [10]–[12]. Intercellular spread begins between 3 and 5 hours post infection [13]–[15]. Following cell-to-cell spread, bacteria are contained within double-membrane vacuoles in a newly infected cell. The bacteria utilize two bacterial phospholipases (PI-PLC and PC-PLC) as well as LLO to escape from spreading vacuoles [1].

Recently it has been suggested that host cells may utilize autophagy as a defense against intracellular pathogens [16]–[18]. Autophagy is a mechanism by which cytoplasmic components, including long-lived proteins and damaged organelles (peroxisomes, ER, and mitochondria) are enveloped within specialized double-membrane-bound vesicles that deliver their cargo to the lysosome for degradation [19]–[21]. It has been hypothesized that basal levels of autophagy occur continuously inside of cells [20]. An increase in autophagic activity can be stimulated by amino acid starvation, hormone signaling, cytokines, TLR stimulation, immunity related GTPases and microbial infection [16]–[18], [20]. However, the mechanism by which substrates are targeted for autophagic degradation is unknown [20].

*L. monocytogenes* has been shown to interact with the host autophagic machinery. Rich et al. (2003) reported that approximately 92% of chloramphenicol treated *L. monocytogenes* were surrounded by double-membrane vacuoles in J774 macrophage-like cells 21 hours post infection. The number of these chloramphenicol-treated bacteria captured by autophagic-like membranes decreased in the presence of autophagy inhibitors [22]. Subsequently, Py et al. (2007) showed that *L. monocytogenes* induces autophagy, as measured by microtubule associated light chain 3 (LC3) lipidation and colocalization of LC3 with intracellular bacteria. Further, Py et al., (2007) provided evidence that *L. monocytogenes* replicate more efficiently in autophagy-deficient (*atg5* ^−/−^) mouse embryonic fibroblasts (MEFs) compared to wild-type MEFs, suggesting that an Atg5-dependent process directly inhibits the replication of wild-type *L. monocytogenes* in mammalian cells. Using similar assays to test for autophagic induction, Birmingham et al. (2007) demonstrated that RAW 264.7 macrophages transfected with LC3-GFP, exhibited colocalization of transfected LC3-GFP with *L. monocytogenes*. However, the authors of this study did not observe that wild-type *L. monocytogenes* grew better in *atg5* ^−/−^ MEFs than in wild-type MEFs. Thus the role of Atg5 in the control of wild-type *L. monocytogenes* replication in cultured transformed MEF cells remains controversial.

Py et al. (2007) and Birmingham et al. (2007) reported that *L. monocytogenes* lacking LLO did not induce autophagy, as measured by LC3I lipidation and colocalization. Further, both ActA and the bacterial phospholipases (PlcA and PlcB) were reported to play a role in escaping autophagy, as measured by LC3I colocalization and bacterial growth in the presence or absence of Atg5 [23], [24]. This suggested that autophagy may play a role in the control of wild-type *L. monocytogenes* replication that is effectively inhibited by the action of bacterial phospholipases and/or actin polymerization. However, this hypothesis has not been directly tested in primary cells. Yano et al. (2008) showed that autophagy in *Drosophila* hemocytes was induced in response to *L. monocytogenes* infection and was dependent upon detection of peptidoglycan by the cytosolic receptor PGRP-LE as well as on bacterial expression of LLO. Lastly, Zhao et al. (2008) revealed that mice lacking Atg5 in macrophages and neutrophils had a slight increase in susceptibility to *L. monocytogenes* as measured by bacterial levels in spleen and liver three days after infection. However, this work did not address whether the role of Atg5 *in vivo* was due to autophagy or an autophagy- independent role of Atg5. Furthermore, Zhao et al. (2008) did not address whether Atg5 controls *L. monocytogenes* replication in primary cells.

We re-evaluated the hypothesis that primary macrophages use autophagy as a defense against invading *L. monocytogenes*. Here we present a detailed cellular study that strongly suggests *L. monocytogenes* induces bacterially targeted autophagy early in infection as a result of phagosomal membrane damage caused by cytolysin expression. However, in contrast to earlier findings in transformed MEFs we report that either autophagy has no role in control of *L. monocytogenes* replication or that an as yet undiscovered bacterial virulence factor effectively circumvents autophagy.

## Results

### *L. monocytogenes* Infection Activates Autophagy

**Figure 1:**
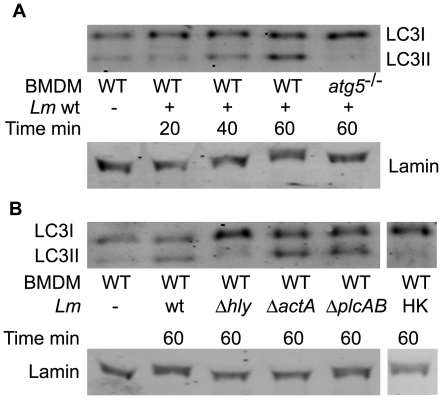
Autophagy is induced by wild-type, Δ*actA*, Δ*plcAB* but not by Δ*hly* or heat-killed *L. monocytogenes*. (**A**) Western blot of LC3I and LC3II in wild-type and *atg5^−/−^* BMDMs infected with wild-type *L. monocytogenes*. All images are from a single gel. (**B**) Western blot of LC3I and LC3II in wild-type BMDMs at 60 minutes post-infection. BMDMs were infected with wild-type, Δ*hly*, Δ*actA*, Δ*plcAB* mutant *L. monocytogenes* as well as heat-killed *L. monocytogenes*. Images taken from a single gel, cropped and combined. Data shown are representative of results obtained in three independent experiments.

To examine the amount of cellular autophagy induced by *L. monocytogenes*, we monitored the conversion of LC3-I to LC3-II in wild-type bone marrow derived macrophages (BMDMs) isolated from C57/B6 mice, infected with *L. monocytogenes* [25], [26]. Activation of autophagy triggers LC3-I lipidation resulting in the conversion of LC3-I to LC3-II, which is measured by a mobility shift on SDS-PAGE [27]. Within 40 minutes of infection with wild-type *L. monocytogenes*, the amount of LC3-II increased compared to uninfected cells (Fig. 1A). Δ*hly* (LLO-minus) *L. monocytogenes* and heat-killed bacteria stimulated almost no LC3-I to LC3-II conversion, as compared to wild-type *L. monocytogenes* over a 60 minute time course of infection (Fig. 1B and data not shown). Therefore, we concluded that while infection alone induced very little autophagy, wild-type bacteria secreting LLO induced increased amounts of autophagy. Further, we found that during a 60-minute infection, the absence of the bacterial proteins ActA and either of the phospholipases did not significantly affect the amount of autophagy activation as measured by LC3-I lipidation (Fig. 1B).

**Figure 2:**
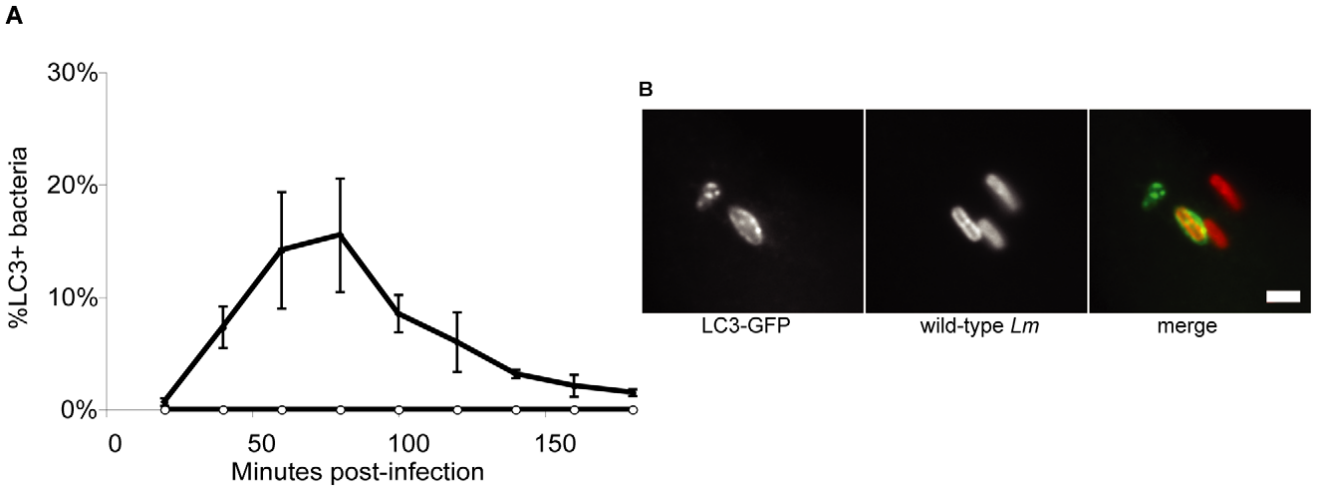
LC3-GFP co-localizes with wild-type *L. monocytogenes*, but does not co-localize with Δ*hly*. (**A**) Percent of BMDMs that contain LC3-GFP, colocalizing with wild-type and mutant (Δ*hly*) *L. monocytogenes* (red) up to three hours post-infection. (**B**) Photomicrographs of LC3-GFP BMDMs 40 minutes post-infection showing colocalization of wild-type *L. monocytogenes* (red) with LC3-GFP. Graphs are a representative of three independent experiments, standard deviations have been included. Images are representative of results obtained in three independent experiments. Bar = 2 µm.

In order to examine the degree of autophagy induction in individually infected cells, we monitored *L. monocytogenes* colocalization with LC3 in infected BMDMs isolated from transgenic mice expressing LC3-GFP [28]. Over the course of three independent experiments, we found that within 60 minutes after infection with wild-type *L. monocytogenes*, approximately 15% of the bacteria co-localized with LC3-GFP, whereas *Δhly* (LLO-minus) bacteria showed no detectable colocalization with LC3-GFP (Fig. 2A,B). Given that infection is asynchronous, these observations represented bacteria at various stages of early infection: entry into cells, within intact phagocytic vacuoles or in the process of escape from the phagosomes. As LC3-GFP did not co-localize with *Δhly L. monocytogenes*, LC3-GFP was either targeting phagosomes damaged by LLO pores or bacteria in the cytosol.

### LLO and PFO Expression by *Bacillus subtilis* Induced Bacteria-Targeted Autophagy

The data presented above support the observation that LLO is required for LC3-GFP colocalization with *L. monocytogenes*. To determine if LLO was the only *L. monocytogenes* determinant of pathogenesis necessary to induce autophagy, we examined the interaction of LC3-GFP in BMDMs with the non-pathogenic Gram-positive bacterium *Bacillus subtilis* engineered to express and secrete LLO or a related cholesterol dependent cytolysin, perfringolysin O (PFO). These modified bacteria have previously been shown to escape into the host cell cytosol, although the efficiency of escape is less than that observed for wild-type *L. monocytogenes* [29], [30].

**Figure 3:**
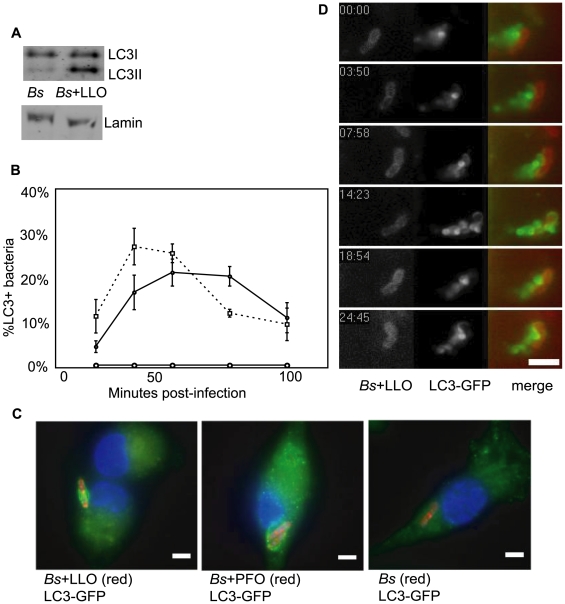
*B. subtilis* expressing cytolysins induced LC3I lipidation and LC3-GPF colocalization in BMDMs. (**A**) Western blot for LC3I lipidation in BMDMs infected with either wild-type *B. subtilis* or *B. subtilis* expressing LLO. (**B**) LC3 colocalization in LC3-GFP BMDMs infected with either wild-type *B. subtilis* or *B. subtilis* engineered to express LLO or PFO. Solid line, closed circles: LC3-GFP BMDMs infected with *B. subtilis* expressing PFO; dashed line, closed squares: LC3-GFP BMDMs infected with *B. subtilis* expressing LLO; solid line, open circles: LC3-GFP BMDMs infected with *B. subtili*. Graphs are a representative of three independent experiments, standard deviations have been included. (**C**) Photomicrographs: LC3-GFP BMDMs 40 minutes post-infection with either wild-type or cytolysin expressing *B. subtilis* (red). Images are representative of results obtained in 3 independent experiments. Bar = 2 µm. (**D**) Stills from time-lapse video microscopy of *B. subtilis* (red) expressing LLO in LC3-GFP BMDMs. Bar = 3.4 µm.

*B. subtilis* expressing LLO induced an increased amount of LC3-I lipidation at 60 minutes post infection as compared to wild-type *B. subtilis* (Fig. 3A). Further, within 40 minutes after infection, *B. subtilis* expressing LLO or PFO co-localized with LC3-GFP equal to or greater than twice as often as wild-type *L. monocytogenes* (Fig. 3B,C). As with *Δhly* (LLO-minus) or heat killed *L. monocytogenes*, wild-type *B. subtilis* did not co-localize with LC3-GFP (Fig. 3B,C). Live video microscopic imaging of cells infected with *B. subtilis* expressing LLO clearly showed LC3-GFP-containing vesicles, approximately 1 µm in diameter, surrounding the bacteria (Fig. 3D).

### LC3 Is Recruited Specifically to LLO-Containing Liposomes in the Absence of Bacteria

These data are consistent with the hypothesis that phagocytic vacuolar membranes damaged by LLO or PFO induce phagosome-targeted autophagy. However, it remained possible that the autophagic machinery was not recruited to the damaged primary vacuole, but rather to the bacterial surface upon exposure to the host cell cytosol.

**Figure 4:**
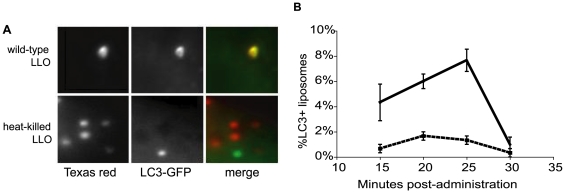
LLO activity is sufficient for autophagy activation, as measured by LC3-GFP colocalization. (**A**) Photomicrographs: liposomes containing, wild-type LLO or heat-killed LLO. Texas Red (TR) = red dye in both wild-type and heat-killed LLO containing liposomes. Images are representative of results obtained in three independent experiments. Bar = 1 µm. (**B**) Percentage of colocalization of LC3-GFP with LLO-containing liposomes over a 30 minute time course. Closed line, closed circles: liposomes containing wild-type LLO; dashed line, closed squares: liposomes containing heat-killed LLO. Data shown are representative of results obtained in 3 independent experiments.

To directly test whether membrane damage induced by LLO was sufficient for autophagy activation during the early stages of infection, we incubated macrophages with liposomes containing either active or heat-killed LLO (HK-LLO) in the presence of Texas Red dextran as a fluid-phase marker [31], [32]. As previously reported, LLO-containing liposomes were phagocytosed, their contents released and the dextran delivered to the cytosol. Of the >300 liposomes counted at each time point over three independent experiments, we found that liposomes encapsulating wild-type LLO had a peak of LC3 colocalization (8%) at 25 minutes post-infection (Fig. 4A,B). The liposomes encapsulating HK-LLO showed less than 2% colocalization with LC3 at the same time point (Fig. 4B). Because only ∼10% of phagosomes harboring LLO-loaded liposomes are competent to rupture (as measured by dye release; KD Lee, personal communication), 8% was not statistically significantly different from this 10% expectation, while 2% was statistically significantly different from both the expected (10%) and observed (8%) values (p>.99 by z-test, using a binomial distribution). These data are consistent with a model in which LLO-mediated membrane damage is sufficient to induce LC3 colocalization in the absence of bacteria.

### Does Autophagy Promote or Prevent *L. monocytogenes* Escape from a Phagocytic Vacuole?

**Figure 5:**
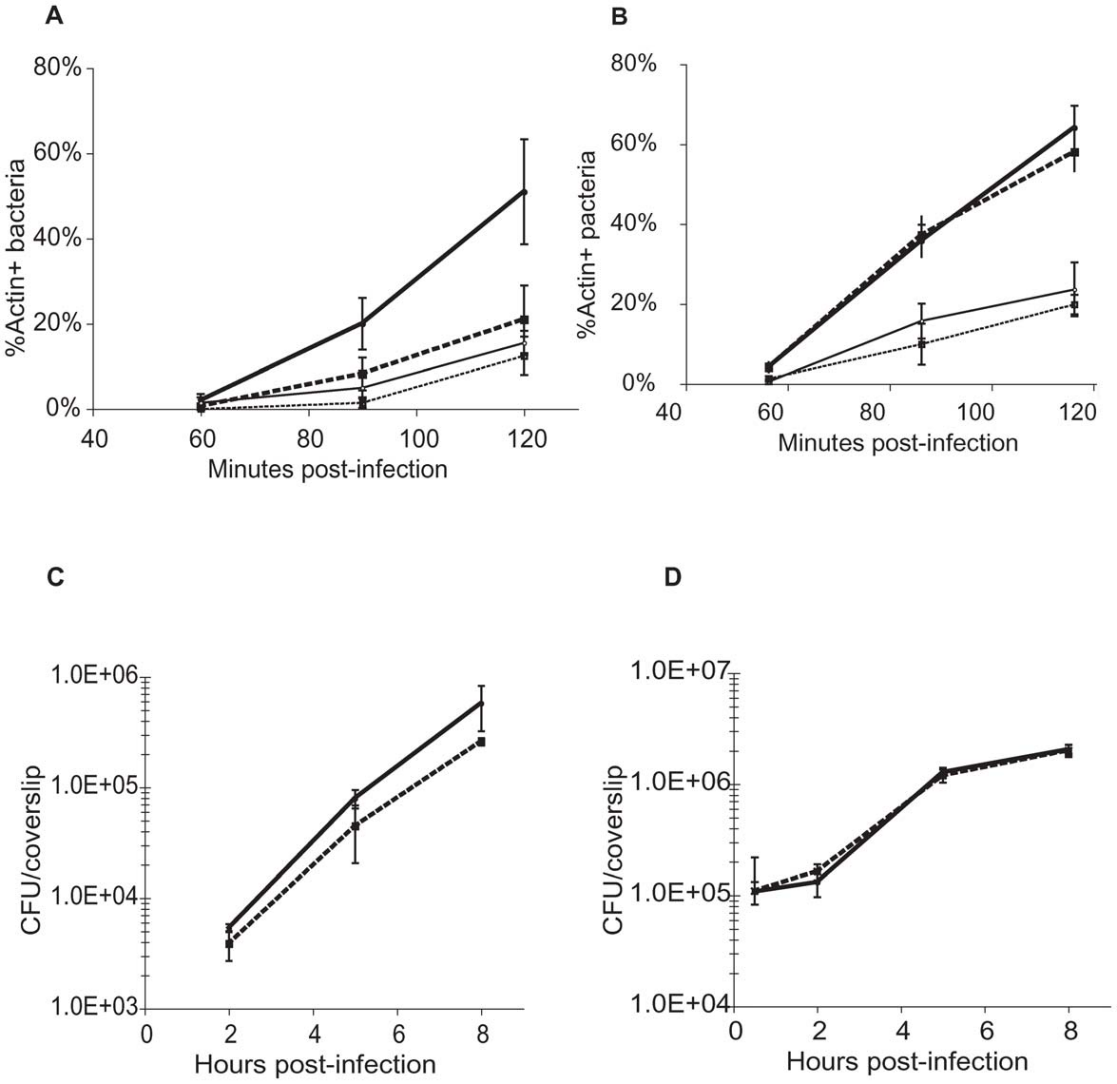
Role of autophagy during *L. monocytogenes* intracellular escape and growth. (**A**) Escape assay in wild-type and *atg5^−/−^* MEFs. Solid line, closed circles: wild-type infected with wild-type *L. monocytogenes*; large dashed line, closed squares: *atg5^−/−^* MEFs infected with wild-type *L. monocytogenes;* solid line, open circles: wt MEF infected with Δ*plcAB* mutant *L. monocytogenes*; small dashed line, closed squares: *atg5^−/−^* MEFs infected with Δ*plcAB* mutant *L. monocytogenes* (**B**) Escape assay in wild-type and *atg5^−/−^* BMDMs. Solid line, closed circles: wild-type MEF infected with wild-type *L. monocytogenes*; large dashed line, closed squares: *atg5^−/−^* MEFs infected with wild-type *L. monocytogenes*; closed line, open circles: wild-type MEF infected with Δ*plcAB* mutant *L. monocytogenes*; small dashed line, closed squares: *atg5^−/−^* MEFs infected with Δ*plcAB* mutant *L. monocytogenes* (**C**) Intracellular growth curves in wild-type and *atg5^−/−^* MEFs. Solid line, closed circles: wt MEF infected with wild-type *L. monocytogenes*; dashed line, closed squares: *atg5^−/−^* MEFs infected with wild-type *L. monocytogenes* (**D**) Intracellular growth curves in wild-type and *atg5^−/−^* BMDMs. Solid line, closed circles: wild-type BMDM infected with wild-type *L. monocytogenes*; dashed line, closed squares: *atg5^−/−^* BMDM infected with wild-type *L. monocytogenes.* Data shown are representative of results obtained in 3 independent experiments.

We hypothesized that a damaged phagosomal membrane was targeted by autophagy and that this interaction could ultimately affect the ability of the bacteria to survive inside of cells and/or escape from a phagosome. The capacity of *L. monocytogenes* to escape from phagocytic vacuoles was examined in both fibroblasts and macrophages that lacked functional autophagy due to the targeted disruption of the essential autophagy gene *atg5* [33], [34]. *L. monocytogenes* escaped at an increased rate from primary vacuoles in the presence of autophagy in MEFs (Fig. 5A). In contrast, the presence of autophagy had no measurable effect on escape rates in bone marrow-derived macrophages (Fig. 5B). Further, contrary to a previous report, bacterial mutants lacking the secreted phospholipases that contribute to vacuolar escape had similar levels of cytosolic entry in both wild-type and *atg5^−/−^* MEFs as well as BMDMs (Fig. 5A, B) [24]. Finally, autophagy did not significantly affect the growth of wild-type *L. monocytogenes* in either MEFs or BMDMs (Fig. 5C, D). Therefore, while LLO stimulated autophagy, there was no observable consequence in bacterial escape or growth in BMDMs. However, the decreased escape observed in *atg5^−/−^* MEFs suggests that under some conditions, autophagy may enhance, rather than prevent, bacterial escape.

## Discussion

There is mounting evidence that autophagy contributes to the innate immune response to microbial pathogens [16]–[18]. Pathogens that grow and or reside in the cytosol, such as: *Shigella flexneri, Francisella tularensis*, and Group A *Streptococci* lyse the host phagosomal membrane, escape into the cytosol and subsequently induce autophagy, as measured by LC3 colocalization, lipidation and electron microscopy [35]–[37]. Bacteria that reside in pathogen-modified vacuoles, such as *Legionella pneumophila, Salmonella* Typhimurium, and *Mycobacterium tuberculosis,* have also been reported to stimulate autophagy, also as measured by the above mentioned techniques [18], [38]–[42]. In both groups of bacteria that induce autophagy, it remains unclear if cytosolic translocation of bacteria and/or bacterial products, and/or phagosomal membrane damage, is the signal that induces autophagy. It is formally possible that all three induce autophagy under some conditions. In *Drosophila*, autophagy is induced by cytoplasmic peptidoglycan sensors, and the signaling pathway involved is independent of the known pathways that induce the expression of antimicrobial peptides [43]. Thus it is possible that *L. monocytogenes* induction of autophagy is multi-step process, with LLO falling upstream of an as-yet-unidentified receptor. However, the data presented here, with LLO- containing liposomes indicate that, while these other putative steps may play a role during bacterial infection, the presence of damaged membranes alone is sufficient to induce LC3 colocalization.

The role of autophagy, if any, during *L. monocytogenes* infection is only just beginning to be appreciated. Previous studies have provided evidence that *L. monocytogenes* triggers autophagy, and that autophagy limits bacterial replication [23], [24], [43]. Further, bacterial mutants lacking the pore-forming cytolysin LLO fail to trigger autophagy [23], [24]. Zhao et al. (2008) recently reported that infection of mice lacking Atg5 in macrophages and neutrophils exhibited a modest increase in *L. monocytogenes* susceptibility.

In this study we confirmed that wild-type *L. monocytogenes*, but not Δ*hly* mutants, triggered autophagy. However, our data from infection of *atg5^−/−^* BMDMs provided no evidence that autophagy had a measurable effect on bacterial escape from phagosomes or on intracellular growth. In contrast, autophagy slightly increased phagosomal escape and intracellular growth in transformed MEFs, which potentially highlights differences in autophagy utilization and/orfunction between various cell types. Additionally, our data provided no evidence that either ActA or bacterial phospholipases affect autophagy as measured by LC3I lipidation and autophagy-restrictive intracellular growth (some data not shown). Finally, by using heterologous expression of LLO and purified LLO encapsulated in liposomes, we showed that LLO activity was not only necessary but sufficient to trigger autophagy. These results are consistent with a model wherein phagosomal membrane damage induces autophagy.

One major difference between our studies and others that studied the interaction between autophagy and *L. monocytogenes* is that we used primary cultures of BMDMs lacking Atg5, while others used transfected RAW macrophage-like cells and *atg5^−/−^* MEFs, respectively [23], [24], [34]. Additionally, we examined phagosomal escape much earlier during infection. The majority of wild-type *L. monocytogenes* escape the vacuole within the first 30 minutes following entry, however Py et al. (2007) assayed escape at four hours post-infection [44]. Since *L. monocytogenes* begin to spread from cell to cell as early as three hours post-infection and phospholipase mutants have a known defect in escape from a secondary vacuole, it is possible that the defect in growth observed by Py et al. (2007) was due to a defect in escape from vacuoles of secondarily infected cells [13], [45], [46]

Autophagy plays a role in cellular homeostasis. For example, smooth ER, excess peroxisomes, damaged mitochondria and rough ER have been shown to be selectively targeted and removed from cells by the autophagic machinery [47]–[50]. We propose that some or all of these organelles are targeted by autophagy due to membrane damage. For example, chemically damaged mitochondria are selectively targeted and removed by autophagy [49]. Selective removal is thought to be due to the disruption of the inner membrane by pores which occurs upon depolarization, a signal which may be similar to pore formation due to cytolysin expression [49]. Our hypothesis is further supported by two reports showing that two pore forming toxins secreted by extracellular pathogens, *Vibrio cholerae* cytolysin (VCC) and the vacuolating cytotoxin from *Helicobacter pylori* (VacA), also induce autophagy in the absence of infection [51]–[53]. Autophagic bodies were identified inside of cells exposed to VCC [52]. Similarly, we noted non-specific induction of autophagy (diffuse puncta formation), when LC3-GFP BMDMs were exposed to increasing concentrations of extracellular LLO (data not shown). Further autophagic induction in response to VacA was blocked when the channel forming activity of the toxin was inhibited [53]. Therefore, autophagy may be a general response to membrane damage, and as a result may have evolved as a mechanism to recognize and target intracellular pathogens.

There are numerous examples of pathogens evading and exploiting host cell biology to promote their pathogenesis. It is likely that the autophagic response to intracellular pathogens is no exception. We suspect that *L. monocytogenes* has acquired the means to avoid or circumvent autophagy by yet undiscovered mechanisms. This is an area of current interest in the lab. We hope to gain a better understanding of how bacterial pathogens have evolved to recognize and evade host cellular processes.

## Materials and Methods

### Bacterial Strains

The wild-type *Listeria monocytogenes* strain used was 10403S [54]. The Δ*hly* (DP-L2161; [55]), Δ*actA* (DP-L3078; [56]) and Δ*plcAB* (DP-L1936; [46]) mutants contained in-frame deletions of the respective genes in the 10403s background.

The wild-type *Bacillus subtilis* strain used was DP-B1066 [30]. It contains a vector-only insertion with no hemolysin. DP-B980 and DP-B1512 contain single copies of *hly* and *pfo* from *L. monocytogenes* and *Clostridium perfringens*, respectively, under the isopropyl-beta-D-thiogalactopyranoside (IPTG)-inducible promoter *p*_spac_ from plasmid pAG58-ble-1 [29]. *B. subtilis* were grown on brain-heart infusion (BHI) agar without antibiotics at room temperature.

### atg5*^−/−^* Mouse Embryonic Fibroblasts

Transformed mouse embryonic fibroblasts (MEFs) were a kind gift from N. Mizushima [36]. The cells were grown in Dulbecco's Modified Eagle Medium (DMEM) and 10% fetal bovine serum (FBS) and passaged a maximum of 5 times when the cells reached approximately 80% confluency. MEFs with a passage of greater than 5 were not used for experimentation.

### atg5*^−/−^* Bone Marrow Derived Macrophages

Wild-type (C57/B6), microtubule light chain associated-green fluorescent fusion protein (LC3-GFP) and *atg5^−/−^* bone marrow-derived macrophages (BMDMs) were isolated from six-to eight-week-old female mice and cultured as previously described [25], [26], [28]. The *atg5^−/−^* mice were generated by mating Cre recombinase lysozyme M mice (Jackson labs, Strain # 004781) with *atg5^flox/flox^* mice [34], [57].

### Infection of Cells

For infection of either BMDMs or MEFs: single colonies of the *L. monocytogenes* were inoculated into 2 mL of BHI broth and grown overnight at 30°C without shaking. A multiplicity of infection (MOI) of ∼5∶1 was used to infect BMDMs (1×10^7^ *L. monocytogenes* and 2×10^6^ BMDMs). An MOI of ∼200∶1 was used to infect MEFs (2×10^8^ *L. monocytogenes* and 1×10^6^ MEFs). BMDMs were washed three times 30 minutes post-infection with room temperature PBS containing calcium and magnesium. Fibroblasts were infected for one hour, washed three times with PBS containing calcium and magnesium, and gentamicin was added at a concentration of 50 µg/mL one hour post-infection. MEFs were washed and gentamicin (50 µg/mL) was added at 60 minutes post-infection.

For infections with *B. subtilis*: *B. subtilis* (from colonies scraped from a plate) were added to 5 mL of BHI broth containing 1 mM IPTG. Cultures were grown at 37°C with shaking to an optical density at 600 nm OD_600_ of 0.8 to 1.2.

For fluorescent labeling of *B. subtilis*: 1 mL of *B. subtilis* cultures at OD_600_ of 0.8 to 1.2 was washed three times in PBS and resuspended in 1 mL PBS containing 0.1 M sodium bicarbonate pH 9.0 and 20 µg Alexa Fluor 594 carboxylic acid, succinimidyl ester (Invitrogen/Molecular Probes). *B. subtilis* were incubated in the labeling reaction gently shaking on ice for 45 minutes. The reaction was washed three times in PBS containing calcium and magnesium and re-suspended in 800 µL PBS containing calcium and magnesium. A range of MOIs from ∼46∶1 to ∼69∶1 of Alexa Fluor 594 labeled *B. subtilis* was used to infect LC3-GFP BMDMs (2.3×10^7^ to 3.45×10^7^ *B. subtilis* and 5×10^5^ BMDMs). The infection was completed in the presence of 1 mM IPTG. At 30 minutes post-infection, cells were washed twice with PBS containing calcium and magnesium and incubated in fresh media containing 1 mM IPTG and 50 µg/mL gentamicin.

### Immunofluorescence

After infection, coverslips containing infected BMDMs were washed once with PBS and fixed in 4% paraformaldehyde for 30 minutes at 37°C. Coverslips containing cells were incubated with blocking buffer (PBS, 0.1% Triton X-100, 2% BSA) for 30 minutes at 37°C. The primary antibodies used were: anti-GFP (combination of two monoclonal murine antibodies, Roche, 1∶200 dilution) and rabbit polyclonal anti-*L. monocytogenes* (Difco, 1∶2,000 dilution). Secondary antibodies used were Alexa-Fluor conjugated goat anti-mouse IgG1 and goat anti-rabbit IgG (Invitrogen, 1∶1000) respectively. Both primary and secondary antibodies were diluted in block buffer and incubated for one hour at room temperature followed by three washes with TBST buffer (25 mM Tris-HCl pH 8, 150 mM NaCl, 0.1% Triton X-100). Coverslips were mounted with Vectashield mounting medium containing DAPI (Vector Laboratories).

### Phagosomal Escape

*L. monocytogenes*-infected cells were stained as described, followed by incubation with a secondary Alexa 488-labeled anti-rabbit polyclonal antibody (Molecular Probes, 1∶2,000 dilution), and F-actin was stained with tetramethylrhodamine B isothiocyanate-phalloidin (Sigma, 1∶1,000 dilution). Both primary and secondary antibodies as well as phalloidin were incubated at room temperature for 60 minutes followed by three washes with TBST buffer. Coverslips were mounted as described above. Bacterial escape was quantified by the number of actin associated (red, phalloidin-positive) fluorescent bacteria over the total number of intracellular green fluorescent bacteria at 30, 60, 90 and 120 minutes post-infection. The percentage of escape was determined by dividing the number of phalloidin-positive (escaped) bacteria by the number of total intracellular bacteria [58]. Numbers were obtained from the average of a minimum of three experiments.

### Western Blotting

An MOI of ∼5∶1 was used to infect BMDMs (1×10^7^ *L. monocytogenes* and 2×10^6^ BMDMs). Cell samples were rinsed once with PBS containing calcium and magnesium and lysed with 2X Laemmli sample buffer (125 mM Tris HCl pH 6.8, 4% sodium dodecyl sulfate, 30% glycerol, 1% β-mercaptoethanol, 0.005% bromophenol blue). The Laemmli buffer was added directly to the infected cells and scraped off the tissue culture treated Petri dishes using a cell scraper. The samples were collected into Eppendorf tubes, boiled for 5 minutes at 95°C and loaded on 12% pre-cast NuPage gels (Invitrogen, Carlsbad, CA). Gels were run in 1 X 3-morpholinopropane-1-sulfonic acid (MOPS) buffer (Invitrogen). Gels were transferred to polyvinylidene fluoride (PVDF) membrane at 15V for 1 hour on a Trans-Blot Semi-Dry Transfer Cell (Bio-Rad, Hercules, CA). The membrane was blocked for 1 hour at room temperature with Odyssey blocking buffer (LI-COR, Nebraska, NE, catalogue no. 929-97301) diluted 1∶1 with PBS. The membrane was incubated overnight at 4°C with mouse monoclonal anti-LC3 antibody (courtesy of R. Kopito and B. Riley, Stanford University) at 1∶500 in blocking buffer containing 0.02% Sodium Azide and 0.1% Tween-20. The membrane was washed three times for 10 minutes with TBST. The membrane was incubated with the LI-COR secondary antibody covalently attached to an infrared-emitting fluorophore IRdye-680 and IRdye-800 (LI-COR Biosciences, Lincoln, NE (goat anti-mouse IgG, 0.5 mg)) for 1 hour at room temperature. The membrane was washed three times for 10 minutes with TBST followed by one wash with 1X PBS. Membranes were scanned at 700 nm or 800 nm using the Odyssey Infrared Imaging System (LI-COR Biosciences), and quantitated using the accompanying software package (Odyssey V 3.0). Anti-gamma-tubulin, mouse monoclonal (clone GTU-88, Sigma catalogue no. T6557) was used as a loading control. Westerns were repeated three independent times.

### LLO-Containing Liposomes

Liposomes (at a concentration of 9 nM) containing either LLO or heat killed LLO (LLO was inactivated by incubation at 70°C for 10 minutes) were prepared as previously described [31]. Liposomes were incubated with BMDMs on coverslips at a 1∶10,000 dilution (ratio of liposome-containing media to cell media) and rinsed with 37°C fresh cell media after three minutes of incubation with the BMDMs. The coverslips containing cells were fixed as described above. At least 300 liposomes were counted per time point and the experiment was repeated three independent times.

### Intracellular Growth Curves

An MOI of ∼5∶1 *L. monocytogenes* was used to infect BMDMs (1×10^7^ bacteria and 2×10^6^ BMDMs). Growth curves were done as previously described [26].

### Microscopy and Image Analysis

Imaging for *L. monocytogenes-*and *B. subtilis*-infected LC3-GFP BMDMs:

Cells were imaged with an Olympus IX71 epifluorescence microscope using the 100X objective for *L. monocytogenes* and 60X objective for *B. subtilis* and 30 frames per time point were randomly selected. Images were collected and color-combined using Metamorph software (Universal Imaging) and subsequently scored for colocalization of *L. monocytogenes* and *B. subtilis* with discrete LC3-GFP containing vacuoles. Images are representative of observed results, repeated in three independent experiments. Images were compiled in Adobe Photoshop software (Adobe, San Jose, CA).

### Imaging for Escape Assays, Liposomes and Live Imaging

Samples were viewed at 600× magnification with a Nikon TE300 inverted microscope. Microscopy was performed on an Olympus IX-70 or IX-80 microscope equipped with a cooled CCD camera. For live imaging, samples were loaded onto a stage heated to 37°C by a fan and antibody staining was not used. Media was changed every ∼60 minutes during long time sequences. Images were collected and color-combined using Metamorph software (Universal Imaging). Large collections of photomicrographs were used for counting cytosolic contents; each graph represents >100 bacteria or liposomes, and three trials for each time point. Figures were compiled in Adobe Photoshop software. Bars are SEM (standard error of mean), where n = 3.
